# Unfractionated and low molecular weight heparin

**DOI:** 10.1186/1753-6561-8-S4-O10

**Published:** 2014-10-01

**Authors:** Marcelo Lima, Helena Nader

**Affiliations:** 1 Departamento de Bioquímica, Disciplina de Biologia Molecular, Universidade Federal do São Paulo, Brazil

## Background

Unfractionated heparin (UFH) is mostly obtained from porcine and bovine mucosa and has been widely used for the treatment and prevention of thrombotic events. It consists of molecular chains of various lengths varying from 2000 to 40,000 Da [1]. Low molecular weight heparins (LMWHs) are smaller chains of UFH that can be obtained, from unfractionated heparin, by various chemical and enzymatic depolymerization processes and since they are produced from natural heparin, they must share structural and functional features with the parent compound, however, the depolymerization process used for their production leads to unique structural and functional characters. Apart from its noble anticoagulant properties, heparin and its derivatives can interact and modulate proteins involved in different biological process such as inflammation [2] and angiogenesis [3]; yet, its mechanism of action are still under debate. Furthermore, heparin broader use is still impaired due to its strong anticoagulant activity and hemorrhagic complications.

## Methods

Employing several physical-chemical analyses, nuclear magnetic resonance spectroscopy, scanning ultraviolet spectroscopy, circular dichroism and chromatographic techniques, coupled to various *in vivo *and *in vitro *biological and functional assays, our laboratory has been, for several decades, in the forefront of heparin and heparin-like structure/function studies as well as their distribution in the animal kingdom.

## Results and conclusions

Over the years we've shown differences in heparin structure, molecular weight and biological activities revealing a tremendous level of variability. As a classical example, the commercial heparins from bovine lung and bovine mucosa differ in the amounts and content of their constituent disaccharides [4]. A systematic study on the structure of some mammalian and invertebrate heparins has shown that all the heparins contain two different basic regions that vary according to the tissue and species of origin. Low and Ultra Low Molecular weight (ULMWHs) heparins have also been extensively characterised, where we've showed that ULMWHs are structurally related to LMWHs; however, their saccharide composition and average molecular weight differ considerably. In general, they possess higher levels of 3-O-sulfated glucosamine residues and higher amounts of unsaturated uronic acid [5].

More recently, the extraction, structural characterization and activity of heparinoids from the Pacific White Shrimp, *L. vannamei*, with repeating structures closely resembling those of heparin, have been reported [3,4]. These heparin-like structures, in contrast to those from mammalian heparin, have low anticoagulant and hemorrhagic potential but, exhibit potentially useful anti-inflammatory and anti-angiogenic activities. Furthermore, against the general accepted view, we've shown that the anticoagulant activity of heparin derivatives are related to the conformational stabilisation of the AT:polysaccharide complex, rather than the induced secondary structural changes in AT. The secondary structural changes induced by active and inactive saccharides were detectable to a high degree of sensitivity by synchrotron radiation circular dichroism (SRCD) but, they did not differ significantly [6]. Altogether, we've been dissecting the mechanism of action of heparin and its derivatives, bringing shedding light on the relationship between structure and function of this class of remarkable important molecules.
